# Treatment of sleep disturbances in trauma-affected refugees: Study protocol for a randomised controlled trial

**DOI:** 10.1186/s13063-017-2260-5

**Published:** 2017-11-06

**Authors:** Hinuga Sandahl, Poul Jennum, Lone Baandrup, Ida Sophie Poschmann, Jessica Carlsson

**Affiliations:** 1 Competence Centre for Transcultural Psychiatry, Mental Health Centre Ballerup, Mental Health Services of the Capital Region of Denmark, Ballerup, Denmark; 2 Danish Center for Sleep Medicine, Department of Clinical Neurophysiology, Rigshospitalet – Glostrup, Copenhagen University Hospital, Nordre Ringvej 57, 2600 Glostrup, Denmark; 3 Mental Health Centre Ballerup, Mental Health Services of the Capital Region of Denmark, Ballerup, Denmark

**Keywords:** Refugee, Trauma, Post-traumatic stress disorder, PTSD, Sleep, Nightmare, Sleep disturbances, Imagery rehearsal therapy, Mianserin

## Abstract

**Background:**

Sleep disturbances are often referred to as a hallmark and as core symptoms of post-traumatic stress disorder (PTSD). Untreated sleep disturbances can contribute to the maintenance and exacerbation of PTSD symptoms, which may diminish treatment response and constitute a risk factor for poor treatment outcome. Controlled trials on treatment of sleep disturbances in refugees suffering from PTSD are scarce. The present study aims to examine sleep-enhancing treatment in refugees with PTSD. We aim to assess if add-on treatment with mianserin and/or Imagery Rehearsal Therapy (IRT) to treatment as usual (TAU) for PTSD improves sleep disturbances. We will study the relation between sleep disturbances, PTSD symptoms, psychosocial functioning and quality of life.

**Methods:**

The study is a randomised controlled superiority trial with a 2 × 2 factorial design. The study will include 230 trauma-affected refugees.

The patients are randomised into four groups. All four groups receive TAU – an interdisciplinary treatment approach covering a period of 6–8 months with pharmacological treatment, physiotherapy, psychoeducation and manual-based cognitive behavioural therapy within a framework of weekly sessions with a physician, physiotherapist or psychologist. One group receives solely TAU, serving as a control group, while the three remaining groups are active-treatment groups receiving add-on treatment with either mianserin, IRT or a combination of both.

Treatment outcome is evaluated using self-administered rating scales, observer ratings and actigraph measurements at baseline, during treatment and post treatment. The primary outcome is subjective sleep quality using the Pittsburgh Sleep Quality Index. Secondary outcome measures are objective sleep length, nightmares, PTSD severity, symptoms of depression and anxiety, pain, quality of life and psychosocial functioning.

**Discussion:**

This trial will be the first randomised controlled trial to examine sleep-enhancing treatment in trauma-affected refugees, as well as the first trial to investigate the effect of IRT and mianserin in this population. Therefore, this trial may optimise treatment recommendations for sleep disturbances in trauma-affected refugees. Based on our findings, we expect to discuss the effect of treatment, focussing on sleep disturbances. Furthermore, the results will provide new information regarding the association between sleep disturbances, PTSD symptoms, psychosocial functioning and quality of life in trauma-affected refugees.

**Trial registration:**

EudraCT registration under the name ‘Treatment of sleep disturbances in trauma-affected refugees – a randomised controlled trial’, registration number: 2015-004153-40, registered on 13 November 2015.

ClinicalTrials.gov, ID: NCT02761161. Registered on 27 April 2016.

**Electronic supplementary material:**

The online version of this article (doi:10.1186/s13063-017-2260-5) contains supplementary material, which is available to authorized users.

## Background

The numbers of forcibly displaced people reached record-high numbers by the end of 2016, with a total of 65.6 million people being forcibly displaced worldwide as a result of persecution, conflict, generalised violence, and/or human rights’ violations. Approximately 22.5 million of these people became refugees. These numbers are currently increasing every year, primarily due to the conflict in Syria [1].

It is estimated that roughly 30% of the world’s refugees suffer from post-traumatic stress disorder (PTSD) and often of a more chronic form, compared to other populations suffering from PTSD [2–4]. Refugees are a heterogeneous group in terms of cultural background and country of origin, but share the experience of being forcibly displaced from their country of origin and in this matter differ from other groups being exposed to traumas, who continue to live under familiar and safe conditions [5]. Refugees differ from populations with single or few traumatic experiences by often having experienced prolonged and repeated traumas pre-migration, during migration and continue to live under post-migration stressors, such as uncertainty about asylum status and temporary residence, concern about their families still unsafe in their home country, cultural and language difficulties, and perceived discrimination and racism [2, 3, 6]. Clinical guidelines for treatment, derived from research on other populations fail to account for the specific circumstances experienced by refugees, and cannot be assumed to apply to trauma-affected refugees in general [5–7].

### Sleep disturbances

Sleep disturbances are often referred to as a hallmark and as core symptoms of PTSD [8–13]. As many as 70–87% of persons suffering from PTSD describe sleep disturbances [8, 10, 11]. In a sample of 752 trauma-affected refugees undergoing psychiatric treatment at Competence Centre for Transcultural Psychiatry (CTP), Mental Health Services in the Capital Region of Denmark, in the period 2008–2012, 99% reported sleep disturbances and recurrent nightmares [14].

Sleep disturbances comprise problems initiating and maintaining sleep, nightmares, early awakening and, consequently, reduced length and quality of sleep. In the following, sleep disturbances refer to the above described and not to the formal diagnoses of insomnia disorder and nightmare disorder in the *Diagnostic and Statistical Manual of Mental Disorders-5* (5th edition; DSM-5) [15].

Standard pharmacological and psychotherapeutic treatments of PTSD often focus primarily on daytime symptoms and rarely examine sleep-related outcomes [9, 13]. Sleep disturbances often persist post treatment. For instance, as many as 48% of patients treated with cognitive behavioural therapy (CBT) for PTSD reported residual insomnia post treatment [12, 13, 16]. Untreated sleep disturbances can contribute to the maintenance and exacerbation of both sleep-related and non-sleep-related PTSD symptoms [8, 9, 12]. Sleep disturbances may also affect the efficacy of first-line PTSD treatment and constitute a risk factor for poor outcome of psychiatric treatment. It has been argued that targeting sleep disturbances in treatment may lead to the alleviation of PTSD symptoms in general and accelerate PTSD recovery [8–12, 17, 18].

Furthermore, sleep disturbances in PTSD are found to be related to increased psychiatric comorbidity, including alcohol use disorder, and poor health status [13].

Sleep disturbances have consequences not only for the individual, by compromising social and vocational functioning and quality of life, but also from a socioeconomic perspective due to reduced productivity, increased absence from work and increased rates of unemployment and early retirement pensions [17]. Previous studies on PTSD found that improved sleep was related to improved global functioning although a causal relationship could not be demonstrated [18].

There is a need for further research on sleep disturbances, nightmares and the relation between improved sleep and global functioning and PTSD symptoms in general [12, 13].

### Treatment of sleep disturbances

A number of studies have been published on psychotherapeutic and pharmacological treatment of sleep disturbances in populations suffering from PTSD, such as war veterans, crime victims and sexual assault survivors [10–13, 18, 19]. However, controlled trials on sleep disturbances in refugees suffering from PTSD are scarce [14].

#### Pharmacological treatment

A range of studies have evaluated pharmacological treatment of PTSD. However, most studies did not evaluate changes in sleep-related outcomes. Reviews on pharmacological treatment of sleep disturbances in PTSD have concluded that antidepressants, benzodiazepines and non-benzodiazepine hypnotics are not beneficial. Only treatment with prazosin (a selective α-1-adrenergic receptor antagonist) has been found effective in more than one randomised controlled trial (RCT) [10, 13, 18]. Prazosin, however, is not marketed in Denmark and is not available for treatment.

In an attempt to relieve sleep disturbances, benzodiazepines and antipsychotics are often prescribed despite side effects and uncertainty about long-term efficiency. Benzodiazepine side effects include development of tolerance, risk of dependence, withdrawal symptoms and cognitive impairment [11]. Antipsychotic drugs have numerous side effects including extrapyramidal symptoms, sedation, glucose dysregulation and weight gain [10, 11, 17, 20].

##### Mianserin as a sleep-enhancing treatment

Mianserin is a noradrenergic and specific serotonergic antidepressant and is known to be well tolerated. Beside its antidepressant capacity, it has anxiolytic and sleep-enhancing capacities. One of its few side effects is sedation which, in this study, is used clinically to enhance sleep. Histamine H_1_-inverse agonist (i.e. strong antihistamine effects) and alfa_1_-antagonist activity is thought to be responsible for the sedative quality [21–23].

In a large-scale trial evaluating treatment of trauma-affected refugees at CTP, treatment with sertraline and add-on treatment with mianserin showed significant improvement of sleep-related items on self-reported ratings (The World Health Organisation-Five Well-being Index (WHO-5), The Harvard Trauma Questionnaire (HTQ) and Hopkins Symptom Check List-23 (HSCL-25)), but due to the study design it was not possible to evaluate whether this was an effect of mianserin [4, 24]. An academic literature review did not identify any other studies in which people suffering from PTSD were treated with mianserin [14]. There is thus a need for further studies.

#### Psychotherapeutic intervention

Due to a high acceptability in patients and a lack of side effects, CBT is recommended as both first-line treatment of primary sleep disturbances, prior to pharmacological treatment, and as first-line treatment of PTSD [11, 17, 25]. Most studies on the psychotherapeutic treatment of PTSD have not evaluated sleep-related outcomes [9, 13]. Current CBT for PTSD does not focus on sleep disturbances [9, 13]. As a consequence, a number of psychotherapeutic interventions targeting sleep disturbances and nightmares have been developed. Cognitive Behavioural Therapy for Insomnia in PTSD (CBT-I) and Imagery Rehearsal Therapy (IRT) have shown promising results [13, 26–28]. A number of treatment manuals exist for particularly IRT, which differ in content and thus complicate comparison and identification of active components of the treatments [13, 26, 29, 30]. Furthermore, there is a lack of studies with an active treatment control group, studies examining predictors of outcome and dismantling studies of treatment components [13, 26, 28].

##### Imagery Rehearsal Therapy as sleep-enhancing treatment

IRT is an adapted CBT, in which the subject rehearses a new and non-disturbing dream to replace the nightmare. IRT has shown promising results in patients suffering from PTSD by improving sleep length and quality and by reducing symptoms of PTSD, but there is a lack of studies on IRT in trauma-affected refugees [14, 31–36]. In 2015, CTP completed a pilot case study on IRT focussing on compliance and acceptability. Based on session attendance, compliance with the methods used, and qualitative interviews about the patients’ experiences with IRT, the pilot study delineated IRT as an acceptable treatment for this population. The pilot study was planned as primarily qualitative and, therefore, the low number of patients (*n* = 5) did not allow for statistical analysis on outcome (Poschmann, I.: Imagery Rehearsal Therapy. Unpublised material). Based on experiences from the pilot study, CTP has developed an IRT manual integrating IRT into CBT supervised by a researcher who previously conducted studies on IRT [37]. The IRT manual is available on the CTP website: www.ctp-net.dk.

### Research objectives and hypotheses

On the background of the two above-mentioned studies from CTP and the absence of relevant or conclusive data on the treatment of sleep disturbances in trauma-affected refugees, the present study aims to examine sleep-enhancing treatment in refugees with PTSD. We hypothesise that add-on treatment with mianserin or IRT to treatment as usual (TAU) will improve sleep quality and sleep length as well as reduce the severity and frequency of nightmares compared to TAU. Furthermore, we hypothesise that add-on treatment with mianserin and IRT to TAU will improve the same parameters more than each add-on treatment alone. We hypothesise that enhanced sleep quality and sleep length will be associated with attenuated PTSD symptoms and with improved observer-rated functioning and self-rated quality of life.

The objectives of this trial are (1) to estimate treatment effects of IRT and mianserin on sleep quality, sleep length and nightmares compared to TAU at CTP (please see description below), (2) to study the relation between enhanced sleep, PTSD symptoms, observer-rated functioning and self-rated quality of life and (3) to examine predictors for positive treatment outcome.

## Methods

**Fig. 1:**
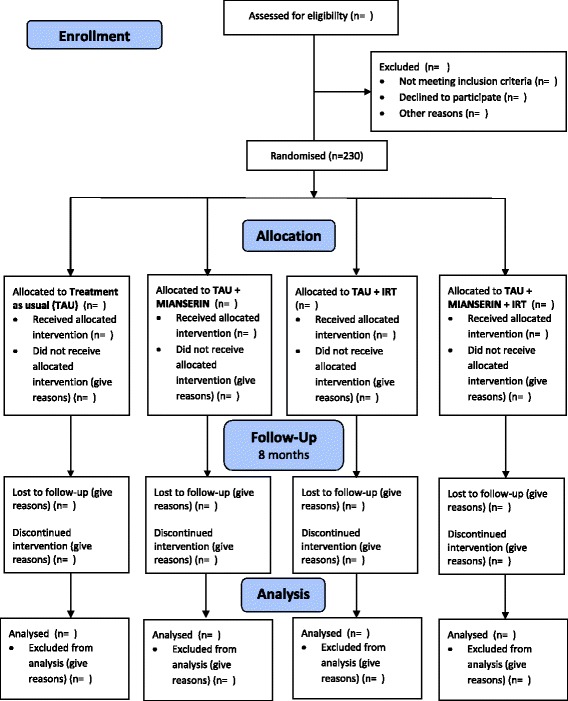
Standard Protocol Items: Recommendations for Interventional Trials (SPIRIT) diagram for enrolment, allocation, follow-up and analysis

The study is a randomised controlled superiority trial with an allocation ratio of 1:1:1:1. The study has a 2 × 2 factorial design. Please see Fig. 1: Standard Protocol Items: Recommendations for Interventional Trials (SPIRIT) diagram for enrolment, allocation, follow-up and analysis. The study will include approximately 230 trauma-affected refugees.

The patients are randomised into four groups. All four groups receive TAU (please see description below); one group receives solely TAU, serving as a control group; the three remaining groups are active-treatment groups receiving add-on treatment with either mianserin, IRT or a combination of both. The course of treatment is divided into two phases: phase 1 (approximately 2 months) and phase 2 (approximately 4–6 months).

For a description of treatment outcome, please see description below and Fig. 1: Schedule of enrolment, interventions and assessments.

### Trial fidelity

Manuals are used in sessions with physicians, physiotherapists and psychologists to establish shared standard procedures. The manuals have been developed by CTP with specific focus on treatment of trauma-affected refugees and are available in Danish on the CTP website: www.ctp-net.dk. All clinicians have been trained in the use of their respective manuals.

In order to determine trial fidelity, patient attendance is registered and after each consultation with a physician, psychologist or physiotherapist, the topics addressed are registered, as well as the methods used during the consultation and whether the patient has completed their exercises between sessions as planned. Whether topics of a social character dominated the session are also registered in order to document obstacles for fidelity to the treatment manuals.

At the end of phase 1 and phase 2 the patients have a blood sample taken to determine the plasma level of mianserin, primarily to determine compliance. Patients who have a concentration of zero in the first blood sample will not be excluded.

### Participants and study setting

Participants are recruited at CTP, a tertiary mental health service outpatient clinic in the Capital Region of Denmark where the trial is also based and data collected. The majority of the trauma-affected refugees referred to CTP are from Afghanistan, Ex-Yugoslavia, Iraq, Iran, Lebanon and Syria. In previous studies, approximately half of the participants had experienced torture and comorbidity of depression was approximately 95% [4].

The majority of the patients speak very little Danish or English. An interpreter is present during 60 and 70% of the sessions. Some of the patients have no, or very little, education and some are illiterate.

### Inclusion criteria

- Adults (18 years or older)
- Refugees or persons who have been family reunified with a refugee
- PTSD pursuant to the *International Classification of Diseases, 10th edition* (ICD-10) research criteria
- Psychological trauma experienced outside Denmark in the anamnesis. Trauma is imprisonment or detention with torture (according to the UN definition of torture) or acts of cruel, inhuman and degrading treatment or punishment. Trauma can also be organised violence, long-term political persecution and harassment, or war and civil-war experiences
- Sleep disturbances/Pittsburgh Sleep Quality Index (PSQI) > 8
- Nightmares/HTQ score on nightmare item of ‘a little’ or higher
- Signed informed consent provided

### Exclusion criteria

- Severe psychotic disorder (defined as patients with an ICD-10 diagnosis F2x and F30.1-F31.9). Participants are excluded only if the psychotic-like experiences are assessed to be part of an independent psychotic disorder and not part of a severe PTSD and/or depression
- Current alcohol or drug use disorder (F1x.24-F1x.26)
- Known neurodegenerative disorder (Alzheimer’s disease, Parkinson’s disease, Lewy-body dementia)
- In need of admission to a psychiatric hospital
- Pregnant and breastfeeding women and women of reproductive age who wish to conceive during the project period
- Allergy towards the active ingredients or excipients in mianserin
- Lack of informed consent provision

### Pre-treatment assessment

All patients referred to CTP undergo a pre-treatment assessment (the content of the assessment is not specific for this trial). The pre-treatment assessment is planned as two to three sessions of approximately 45 min with a physician and consists of recording of the trauma history, the migration process, sociodemographic characteristics (education, job, marital status, family and housing), somatic and psychiatric medical history, handedness, somatic examination as well as a clinical assessment. Standardised diagnostic tools, such as part of Schedules for Clinical Assessment in Neuropsychiatry (SCAN) [38] and the ICD research criteria, are applied in the interview. The informed consent is obtained from patients in this pre-treatment assessment.

### The intervention and the course of treatment

All four groups receive TAU (please see description below). The groups receive the following treatment: (1) TAU, (2) TAU and add-on treatment with mianserin administered in sessions with a physician, (3) TAU and, in phase 2, add-on treatment with IRT integrated in manual-based CBT in sessions with a psychologist, (4) TAU and, in phase 1 and 2, add-on treatment with mianserin administered in sessions with a physician, and in phase 2 add-on treatment IRT integrated in manual-based CBT in sessions with a psychologist.

Phase 1 consists of weekly sessions of 45 min with a physician (six sessions) and weekly sessions with a physiotherapist (six sessions). Phase 2 consists of monthly sessions with a physician (four sessions), weekly sessions with a psychologist (16 sessions) and sessions with a physiotherapist (two sessions).

#### Treatment as usual (TAU)

Treatment as usual (TAU) for trauma-affected refugees at CTP is an interdisciplinary treatment approach covering a period of 6–8 months with medicine according to standard at CTP (best clinical practice in the field), physiotherapy, psychoeducation (including sleep hygiene education and relaxation techniques) and manual-based CBT.

Interpreters are present in sessions if needed and during conduction of ratings as required. The interpreters are all affiliated with CTP and are experienced in interpreting questionnaires, psychotherapy and psychoeducation consultations.

##### Physician

Pharmacological treatment follows an algorithm in the physician’s manual.

According to this manual, sertraline is first-choice antidepressant, venlafaxine second-choice antidepressant and imipramine third-choice antidepressant. In a previous study at CTP, the effect of sertraline and venlafaxine on sleep disturbances were found to be equal [39, 40]. When steady state is reached, we aim to keep co-medication constant, but any clinically necessary changes are allowed.

Many patients who are referred to CTP already receive psychopharmacological treatment. When possible, patients gradually phase out other psychopharmacological treatments, and treatment according to algorithm is initiated. Co-medication is allowed and all changes, which are clinically necessary, are allowed and will be controlled for in the analysis if necessary.

In addition to the pharmacological treatment, patients receive psychoeducation in an individually adjusted course, in which relevant topics are addressed, such as knowledge of the disorders (PTSD, depression and anxiety), sleep (sleep hygiene education and relaxation techniques), pain, concentration and memory as well as exercise and lifestyle.

##### Psychologist

Previously, at CTP, different psychotherapy manuals have been used, based on various combinations of Trauma-Focussed CBT (TF-CBT), Acceptance and Commitment Therapy (ACT), mindfulness, stress management (SM) and cognitive restructuring. On the basis of the experience with these previous manuals, a new manual has been prepared based on CBT, but adapted to the target group. In the following, therapy given on the basis of this updated manual will be referred to as manual-based CBT.

A preliminary psychological assessment is carried out prior to manual-based CBT by the psychologist in one or two sessions in order to assess reflective functioning and plan the individual course of therapy.

##### Physiotherapist

The focus of the sessions is on the ability to relax by following instructions in relaxation techniques and the ability to create a mentally calm place. The exercises include instructions in resting positions, body scans, and other relaxation techniques combined with breathing exercises. This is supported by exercises in reducing existing pain. The sessions also include psychoeducation on sleeping habits concerning sleep and daily routines.

##### Trial medication – mianserin

Mianserin is initiated at 10 mg daily per os. The dose can be increased gradually to a maximum dosage of 30 mg daily per os adjusted according to effect and side effects.

At each session with the physician, the patients are asked if they have taken their medication as prescribed, and the dose of mianserin is registered.

Compliance is monitored by measuring the plasma concentration.

##### Imagery Rehearsal Therapy (IRT)

IRT is integrated in six sessions of manual-based CBT.

The sessions consist of psychoeducation on disturbing dreams, nightmares and sleep as well as exercises in cognitive restructuring and imagination, enabling the patient to transform the disturbing dream or nightmare into a new and non-disturbing dream. All psychologists have been trained in this specific method described in detail in the IRT manual.

### Outcome

**Fig. 2:**
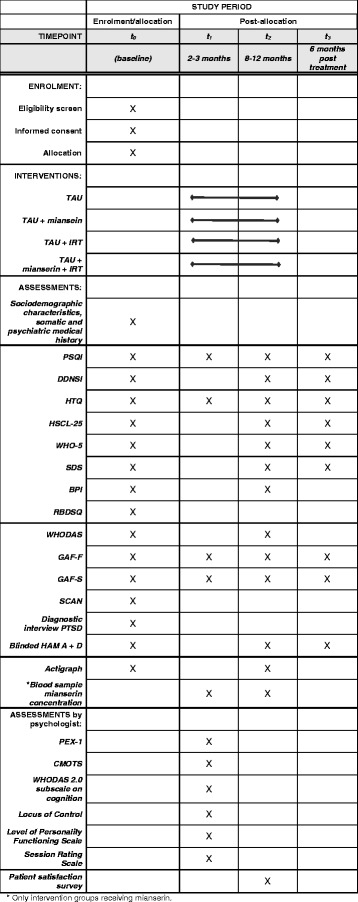
Schedule of enrolment, interventions and assessments

Treatment outcome is evaluated using both self-administered rating scales and observer ratings (please see Fig. 2: Schedule of enrolment, interventions and assessments). All self-administered rating scales have been translated into the relevant languages. The rating scales applied have been validated in several languages as well as cultural settings.

The primary outcome in this trial is sleep quality measured on the Pittsburgh Sleep Quality Index (PSQI) [41, 42]. Secondary outcome measures are nightmares (the Disturbing Dreams and Nightmare Severity Index (DDNSI) [43]), sleep length (actigraphy), PTSD severity (Harvard Trauma Questionnaire (HTQ) [44]), symptoms of depression and anxiety (Hopkins Symptom Check List (HSCL-25) [45] and blinded Hamilton depression and anxiety rating scales (HAM-A and HAM-D) [46]), pain (Brief Pain Inventory short form (BPI) [47, 48]), quality of life (WHO-5 [49]) and level of functioning (Sheehan Disability Scale (SDS) [50], Global Assessment of Functioning – Symptoms *(*GAF-S), and Functioning (GAF-F) [51] and The World Health Organisation Disability Assessment Schedule (WHODAS 2.0) [52] (please see Fig. 2: Schedule of enrolment, interventions and assessments).

Patients complete self-administered rating scales pre-treatment (baseline), between phase 1 and 2, and post treatment. Both psychologists and physicians take part in regular training sessions to ensure high interrater reliability in relation to observer ratings.

Blinded HAM-A and HAM-D observer ratings take place at baseline, post treatment and at 6 months’ follow-up post treatment. HAM-A and HAM-D ratings are carried out by assessors blinded to intervention group and pre or post treatment. The outcome assessors carrying out HAM-A and HAM-D are trained medical students, who participate in regular training sessions to ensure high interrater reliability.

As a supplement to the subjective measures of sleep, all patients are equipped with an actigraph to measure objective sleep length for a period of 2 weeks at baseline and for 2 weeks post treatment. The actigraphic recordings are supplemented by sleep logs where patients register subjective sleep length, sleep quality and nightmares.

### Primary outcome measure

- *The Pittsburgh Sleep Quality Index (PSQI)* is an internationally applied and thoroughly validated self-administered rating scale assessing sleep quality and the severity of sleep disturbances. PSQI consists of 19 items and measures seven components of sleep: sleep quality, sleep latency, sleep duration, habitual sleep efficiency, sleep disturbances, use of sleeping medications, and daytime dysfunction. The component scores each has a range of 0–3 points and they are added to yield one global PSQI score (range of 0–21 points) which distinguishes good sleep (PSQI total score ≤ 5) from poor sleep (PSQI total score > 5) [41, 42].

### Secondary outcome measurements

#### Self-administered rating scales

- The *Disturbing Dreams and Nightmare Severity Index (DDNSI)* is an internationally applied and thoroughly validated self-administered rating scale assessing frequency and severity of nightmares [43]
- *The Harvard Trauma Questionnaire (HTQ)* is an internationally applied and thoroughly validated self-administered rating scale assessing the severity of PTSD symptoms. The first 16 questions of the HTQ, Part IV (symptoms part) are used covering all PTSD criteria in accordance with ICD-10 as well as DSM-IV [44]
- The *Hopkins Symptom Check List-25 (HSCL-25)* is an internationally applied and thoroughly validated self-administered rating scale with 25 questions assessing the severity of anxiety (10 questions) and depression (15 questions) symptoms. This is a short version of the Symptom Checklist-90 (SCL-90) [45]
- *The World Health Organisation-Five Well-being Index (WHO-5)* is a self-administered questionnaire evaluating quality of life, consisting of five questions. The questionnaire has been used to assess quality of life in a number of psychiatric diagnostic groups [53, 54]. In addition, the scale has been used to assess overall treatment effects in the field of psychiatry [49]
- The *Sheehan Disability Scale (SDS)* is a self-administered rating scale measuring functional impairment with regard to family, work and social network using three visual analogue scales from 0 to 10. Evaluation of the scale has shown that it is sensitive to treatment effects in psychiatric patients [50, 55]
- The *Brief Pain Inventory short form (BPI)* is a self-administered rating scale assessing severity of pain, impact of pain on daily functioning, location of pain, pain medications and amount of pain relief in the past 24 h or the past week. The BPI has been through psychometric and linguistic validation in more than 20 languages [47, 48]

#### Observer ratings

- *Global Assessment of Functioning – Symptoms (GAF-S) and Functioning (GAF-F)* are numeric observer rating scales used to assess the degree of psychiatric symptoms and global functioning in adults [51]
- *The World Health Organisation Disability Assessment Schedule (WHODAS 2.0)* is an observer rating scale used to measure health and disability across cultures [52]
- The *Hamilton anxiety and depression scales (HAM-D and HAM-A)* are observer rating scales assessing depression and anxiety using semi-structured interviews. The scales have been used widely in psychiatric research including on trauma-affected refugees [39, 53, 56–58]
- *The CTP Predictor Index* was developed in relation to a previous trial at CTP to rate the psychosocial resources of the patients [39, 59]. In this trial, the index presented promising results as a predictor of treatment outcome. The index was subsequently adjusted according to the experiences in this previous trial by dividing one predictor (employment status) into three predictors (employment status, daily activity and patient-perceived economic situation). Furthermore, one predictor (education) was removed. The rating consists of 16 potential predictors: five rated by the physician, five rated by the psychologist, and six rated by the social worker. The predictors concern the patients’ past, chronicity of mental health problems, pain, treatment motivation, prerequisites for engaging in psychotherapy as well as social and economic situation. Each potential predictor is rated according to pre-defined criteria. The physician, psychologist and social worker complete the index during their first session with the patient [39, 40]

#### Sleep and dream variables

- An *Actigraph* is a watch-like device worn on the wrist measuring movement and amount of light. The actigraphic recording can be used to distinguish between periods of being awake and periods of sleep. The actigraph measurements can be used to estimate total sleep duration, sleep duration in daytime and daily routines [60, 61]
- The *REM Sleep Behaviour Disorder Screening Questionnaire (RBDSC)* is a self-administered questionnaire with 10 items, assessing sleep behaviour using ‘yes’ or ‘no’ questions. It has been developed to assess the most prominent clinical features of REM Sleep Behaviour Disorder [62]

#### Psychologist assessment

- The *Psychotherapy Preferences and Experiences Scale (PEX1)* is a self-administered rating scale measuring preferences and experiences of psychotherapeutic interventions. It consists of 25 questions [63]
- The *Client Motivation for Therapy Scale (CMOTS)* is used as a semi-structured interview assessing motivation for therapy [64, 65]
- *The World Health Organisation Disability Assessment Schedule (WHODAS 2.0)* subscale on cognition is an observer rating scale with six questions used to assess concentration, memory, problem solving, learning and communication [52]
- *Locus of Control (LOC)* is used in an adapted scale to assess the degree to which people believe that they have control over their own lives [66]
- The *Level of Personality Functioning Scale (LPFS)* is an observer rating scale used to assess personality-related functioning with reference to the normal personality [15]
- The *Session Rating Scale (SRS V.3.0)* is a self-administered rating scale used to evaluate the session [67]

#### Mental state assessment

- In each session with a physician, the patient’s condition is assessed through a standardised clinical examination covering 14 parameters such as mood, suicidality and psychotic symptoms

#### Patient satisfaction survey

After the evaluation interview, the patient fills in a questionnaire on satisfaction with treatment. The questionnaire has 15 questions. Each item assesses to which degree respondents were satisfied with different aspects of the treatment program.

### Other variables measured to evaluate trial medication adverse events

The patients are asked about adverse events (AEs) in each session with a physician. Adverse events are registered. From the patient being randomised and until the patient stops participation in the trial, Each serious adverse event (SAE), serious adverse reaction (SAR) and suspected unexpected serious adverse reaction (SUSAR) is registered in accordance with definitions and current legislation by the Danish Medicines Agency. Orifarm summary of product characteristics is used for mianserin as a reference document to assess whether an AE or adverse reaction is unexpected. Planned hospitalisation is not considered a SAE. In addition, all discomfort in connection with psychotherapy is registered.

### Randomisation

The actual randomisation procedure is carried out as follows: the randomisation sequence was computer generated by the Department of Biostatistics at the University of Copenhagen with allocation sequence with block size unknown to the investigator. This department is not otherwise involved in the research project. The Department of Biostatistics at the University of Copenhagen drew up a randomisation list. A person, who was neither associated with the treatment nor the management of the trial converted the randomisation list into sealed, sequentially numbered envelopes. The randomisation list is kept at the Department of Biostatistics.

Two secretaries not involved in the daily work at CTP manage the envelopes.

When a patient is found to be eligible for inclusion and has signed informed consent, the physician contacts the secretary mentioned above. The secretary opens the envelope and informs the physician about the assigned intervention group. The physician immediately notes the assigned intervention group on the patient record. The secretary notes the date of birth of the randomised patient on a randomisation list in order to enable backtracking of the randomisation process. In the following session, the physician informs the patient about the assigned intervention.

The randomisation is stratified by gender.

### Blinding

Blinding of patients and clinicians is not possible due to the different nature of the treatment interventions. However, blinded raters perform the HAM-A and HAM-D ratings pre and post treatment.

Data assessment and data analysis will be performed blinded.

### Statistics

#### Power calculations and size of material

The primary outcome is PSQI. In previous trials, the Minimal Clinically Important Difference (MCID) on PSQI was considered 2.5 scale points. This trial aims to detect a clinically important difference between TAU and add-on treatment and not merely a statistically significant difference and hence MCID was set to 2.5 scale points on the PSQI and the within-groups standard deviation was set to 3 scale points [68]. With a power of 90% and alpha 0.05 we estimated a sample size for each group of 32 and a total of 128. Based on the completion rate in previous trials at CTP, 75–80% of the patients were estimated to complete the treatment [53, 59]. Due to the expected large dropout, a formula (*k* = 1/(100% − dropout%)^2^) calculating the enhanced number of patients needed in each group was used. We increased the number of patients included with a factor *k* = 1/(100% − 25%)^2^ = 1.78 × 128 and consequently estimated a total sample size of 228 patients.

The trial will be stopped when approximately 230 patients have completed the trial.

#### Dropout analysis

Dropout analysis is based on the patients who attend the pre-treatment assessment.

The patients will be compared with the patients who were excluded at the pre-treatment assessment on a number of dimensions in order to identify possible systematic selection bias.

The patients included in the trial, but who eventually drop out and do not complete the trial, will be analysed using intention-to-treat analysis. In addition, completer analyses will be carried out.

#### Data processing

The study population is expected to score very highly on PSQI, which means that even a significant change will not necessarily bring the score under cut-off values. Hence, the primary endpoint PSQI should be analysed as a quantitative variable. Most outcomes are suitable for linear regression, factorial 2 × 2 analysis of variance (ANOVA) analysis with and without relevant covariates and Full Information Maximum Likelihood (FIML) analyses (to handle missing data).

In order to analyse changes over time, we will calculate differences between baseline and follow-up scores. We will analyse potential predictors of outcome by linear regression and by logistic regression.

We plan a number of explorative subgroup analyses including age, gender, average score on PSQI and HTQ in an attempt to isolate predictors of positive outcome.

Actigraphy data will be analysed using standardised software-implemented algorithms of sleep continuity parameters as well as calculation of more specific markers of circadian stability.

### Publications

Positive as well as inconclusive or negative results will be published.

Three publications are planned corresponding to the three purposes of the study: (1) treatment effect on sleep disturbances comparing mianserin, IRT and a combination of both, (2) the relation between enhanced sleep, PTSD symptoms, physician-evaluated functioning and patient-evaluated quality of life and (3) predictors of treatment outcome.

If the results cannot be published in a journal, they will be published at www.clinicaltrials.gov or www.clinicaltrialsregister.eu.

Trial results will be communicated to patients via letter.

The Vancouver rules for authorship will be followed. There will be no use of professional writers.

## Discussion

The presentation of the current study protocol ‘Treatment of sleep disturbances in trauma-affected refugees – a randomised controlled trial’ is in accordance with the Standard Protocol Items: Recommendations for Interventional Trials (SPIRIT) 2013 Statement for clinical trial protocols; please see Additional file 1.

This trial will be the first randomised controlled trial to examine sleep-enhancing treatment in trauma-affected refugees as well as the first trial to investigate the effect of IRT and mianserin on sleep disturbances, PTSD symptoms, functioning and quality of life in trauma-affected refugees. No evidence-based treatment of sleep disturbances in this population exists. Therefore, this trial may optimise treatment recommendations for sleep disturbances in trauma-affected refugees. Furthermore, the results will provide new information about the association between sleep disturbances, PTSD symptoms, psychosocial functioning and quality of life in trauma-affected refugees.

A limitation to the trial is that neither the patient nor the clinicians are blinded to treatment groups. Furthermore, the primary outcome measure, PSQI, is a self-administered rating scale and this can be a limitation due to over- or under-estimation of treatment effect.

The treatment outcome, covering both subjective and objective measures, allows for high-quality analysis. The risk of biased effect estimates is reduced by blinded outcome assessors for HAM-A and Ham-D and intention-to-treat analysis. Furthermore, the blinded 6-month follow-up is a strength of the trial.

### Trial status

Screening for patients for this trial began on 15 March 2016. The first patient was included on 12 May 2016. Inclusion is expected to continue until 31 December 2017.

## Additional file

Additional file 1: SPIRIT 2013 Checklist: recommended items to address in a clinical trial protocol and related documents. (DOC 123 kb)

## Abbreviations

AE: Adverse events
ANOVA: Analysis of variance
AR: Adverse reactions
BPI: Brief Pain Inventory short form
CBT: Cognitive behavioural therapy
CBT-I: Cognitive Behavioural Therapy for Insomnia in PTSD
CMOTS: Client Motivation for Therapy Scale
CTP: Competence Centre for Transcultural Psychiatry
DDNSI: The Disturbing Dreams and Nightmare Severity Index
DSM: *Diagnostic and Statistical Manual of Mental Disorders*
GAF- S and GAF-F: Global Assessment of functioning – Symptoms and functioning
GCP: Good Clinical Practice
HAM-A and Ham-D: Hamilton anxiety and depression scales
HSCL-25: Hopkins Symptom Checklist-25
HTQ: Harvard Trauma Questionnaire
ICD-10: *International Classification of Diseases, 10th edition*
IRT: Imagery Rehearsal Therapy
Manual-based CBT: manual-based cognitive behavioural therapy
PEX1: Psychotherapy Preferences and Experiences Scale
PSQI: Pittsburgh Sleep Quality Index
PTSD: Post-traumatic stress disorder
RBDSC: REM Sleep Behaviour Disorder Screening Questionnaire
SAE: Serious adverse event
SAR: Serious adverse reaction
SCAN: Schedules for Clinical Assessment in Neuropsychiatry
SDS: Sheehan Disability Scale
SRS: Session Rating Scale
SUSAR: Suspected unexpected serious adverse reaction
TAU: Treatment as usual
WHO-5: Well-being Index-five
WHODAS: The World Health Organisation Disability Assessment Schedule
